# Optimal Path Planning for Selective Waste Collection in Smart Cities

**DOI:** 10.3390/s19091973

**Published:** 2019-04-27

**Authors:** María-Victoria Bueno-Delgado, José-Luis Romero-Gázquez, Pilar Jiménez, Pablo Pavón-Mariño

**Affiliations:** 1 Telecommunication Networks Engineering Group (GIRTEL), Department of Communications and Information Technologies, Technical University of Cartagena, 30202 Cartagena, Spain; josel.romero@upct.es (J.-L.R.-G.); pablo.pavon@upct.es (P.P.-M.); 2 E-lighthouse Network Solutions, 30203 Cartagena, Spain; 3 Transportation Engineering Group, Department of Civil Engineering, Technical University of Cartagena, 30202 Cartagena, Spain; pilar.jimenez@upct.es

**Keywords:** waste collection, path optimization, Net2Plan, software platform, smart city, smart container, open-source

## Abstract

Waste collection is one of the targets of smart cities. It is a daily task in urban areas and it entails the planning of waste truck routes, taking into account environmental, economic and social factors. In this work, an optimal path planning algorithm has been developed together with a practical software platform for smart and sustainable cities that enables computing the optimal waste collection routes, minimizing the impact, both environmental (CO_2_ emissions and acoustic damage) and socioeconomic (number of trucks to be used and fuel consumption). The algorithm is executed in Net2Plan, an open-source planning tool, typically used for modeling and planning communication networks. Net2Plan facilitates the introduction of the city layout input information to the algorithm, automatically importing it from geographical information system (GIS) databases using the so-called Net2Plan-GIS library, which can also include positions of smart bins. The algorithm, Net2Plan tool and its extension are open-source, available in a public repository. A practical case in the city of Cartagena (Spain) is presented, where the optimal path planning for plastic waste collection is addressed. This work contributes to the urban mobility plans of smart cities and could be extended to other smart cities scenarios with requests of optimal path planning.

## 1. Introduction

Many cities around the world pursue becoming smart cities, making use of information and communication technologies (ICTs) to tackle those problems emerging in dense urban areas: pollution, traffic jam, waste management, recycling or sustainability policies [1,2,3]. 

Waste collection is one of the targets of smart cities and it has been gaining attention in recent years [4,5]. Waste collection is a daily task in urban areas that entails the planning of waste truck routes, taking into account environmental, economic and social factors. Routes should be planned to avoid traffic jams or acoustic impacts, to minimize fuel costs and CO_2_ emissions, while maximizing the amount of waste collected per route. The latter entails knowing the level of waste in containers. Some solutions have been proposed using Internet of Things (IoT) devices to measure the fill level in containers and to send this data through the Internet to a server for decision-making [6,7,8,9,10].

In an ideal scenario, the truck routes planned for waste collection should not be static, but adapted to the dynamism of cities, that is, street cuts, re-arrangements of street directions, traffic variation or the varying fill level of waste containers, among others. The latter could vary according to the day or by unexpected changes, for instance the opening of shopping centers, restaurants, etc. 

In this work, an optimal path planning algorithm has been developed with the aim of computing the optimal waste collection routes in cities with different time schedulers (by hours, days, weeks, etc.) minimizing the environmental and socioeconomic impact. The algorithm solves an integer linear programming (ILP) problem, fed by:

- City layout given by a Geographical Information System (GIS) database, that includes information of roads, location of containers in streets.
- Number of trucks available and their capacity.
- Capacity and filling level in containers collected by smart devices (e.g., sensors with general packet radio service (GPRS) module) attached to waste containers.
- Fill level threshold, which fixes the set of containers that must be emptied.

The algorithm can be also fed by those constraints imposed by the scenario under study (street cut, daily/nightly schedule, etc.).

- Using the above information, the algorithm jointly calculates the optimal number of trucks to use and the path they have to follow for collecting the waste of those containers with a fill level equal or higher than the threshold, minimizing the routes (total distance in km), which are directly related to fuel consumption, amount of CO_2_ emissions and waste collected.

Our proposal has been implemented as a Net2Plan [11,12] algorithm. Net2Plan is an open-source tool designed for the modeling and planning of communication networks. The ILP inside our algorithm is solved using JOM (Java Optimization Modeler) [13], a free and open-source Java library for modeling optimization programs and interfacing with solver libraries that produce numerical solutions for them. A use case is presented in the city of Cartagena (Spain), where the optimal path planning for plastic waste collection is solved. The city layout information has been imported into Net2Plan using the Net2Plan-GIS extension [14]. Net2Plan-GIS enables the management of data to and from GIS databases. 

The algorithm proposed in this work is free available in a public repository [15] as well as Net2Plan [16] and Net2Plan-GIS [17]. This work contributes to the urban mobility plans of smart cities for optimal waste collection and could be extended to other smart cities scenarios with requests of optimal path planning: optimal routes for bicycle lines, optimal routes of municipal buses, etc. 

The rest of the paper is organized as follows: Section 2 summarizes the related work. Section 3 explains the algorithm developed. Section 4 describes the case study. Section 5 presents the parameters formulation. In Section 6 the results are discussed. Finally, Section 7 concludes.

## 2. Related Work

In the last few decades, several studies have been conducted addressing the so-called vehicle routing problem (VRP) or capacity VRP (CVRP) [18]. VRP/CVRP are aimed at computing the shortest routes (time, distance, cost) for a group of vehicles, meeting some requirements, i.e., routes start and end in the same point, streets are traversed only once, a set of streets must be visited, vehicle capacity constraints, etc. 

Some works in the scientific literature address the VRP/CVRP problem with algorithms that solve an ILP or mixed-integer linear programming (MILP) problem [9,19,20]. Despite their accurate results, the computational complexity (nondeterministic polynomial hard problems or NP-hard problems) makes them unaffordable for problem instances of medium-large size. To overcome this barrier many heuristic and meta-heuristic approaches were proposed [21,22,23,24,25,26,27,28]. Some examples are those based on nearest neighborhood search [21], ant colony optimization [22], genetic algorithms [23], particle swarm optimization [24] or Tabu search [25]. 

From the above algorithms, only a few have other features that add the flexibility required in the waste collection in real cities. That is, to permit setting as input in the algorithm different start and end points in the routes, to set the maximum number of times a street can be traversed by a vehicle, or to jointly decide the number of trucks to use and the route followed by each of them. Our work covers also these aspects. In addition, our scheme is integrated into an open source software framework that gives practical access to input data from GIS databases. This has been used to conduct a realistic use case, where we study the benefits of utilizing smart bins and assess the effect of applying different thresholds to decide when to collect the bins. 

Table 1 summarizes these features for the set of algorithms reviewed in this section and the algorithm proposed in this work (***). The comparison shows that most of the algorithms reviewed are based on heuristic or metaheuristic models and seek to minimize the length of the routes. Only in [24] the algorithm maximizes the total waste collected, while in [25] authors present a bi-objective model with the aim at minimizing the total cost of transport, which depends on the vehicle used and length of routes, while maximizing the quality of service, given by a function that measures the accumulation of residues in streets. The algorithm proposed in this work aims at minimizing the total cost of a waste collection decision, which depends on (i) variable costs growing linearly with the length of the routes and (ii) fixed costs, depending on the number of trucks used. 

Regarding the use as input data from GIS or smart bins, only the works in [9,21] and the algorithm proposed in this work, make use of both technologies. Algorithms constraints are also reviewed to show if they permit to configure the times a vehicle can pass through a street. Finally, we identify those algorithms that provide as output in the algorithm the number of trucks to use. 

The algorithm proposed in this work is aimed at minimizing cost of waste collection and includes all the features reviewed. Although it is modeled as an ILP, the computational complexity is low because the input data in the algorithm (capacity of vehicles, capacity of bins and threshold) help to reduce the number of decision variables. In summary, the main contributions of this work are:

- The proposal of an optimization algorithm that jointly optimizes the number of trucks to use and their routes, also permitting setting constraints on the number of times a street can be traversed, e.g., to limit the acoustic impact in the neighborhood.
- Our algorithm integrates into the Net2Plan-GIS open-source framework. This facilitates introducing GIS information into the problem, applying the algorithm in real-life use cases. In addition, the framework automatically computes a number of economic and environmental performances like fuel consumption or CO_2_ emissions.
- To showcase the practical interest of our proposal, we present a realistic use case in the city of Cartagena (Spain), where we assess the benefits of using smart bins in the waste collection in a part of the city. Our algorithm is used to evaluate the effects of applying different fill level thresholds for deciding when to collect a bin and the benefits of optimizing also the number of trucks to be used.

## 3. Optimal Path Planning Algorithm for Waste Collection

An ILP problem has been implemented as a Net2Plan algorithm [12,13] to solve the path planning for waste collection in a city. The problem is formulated to solve how many trucks have to be used to collect the waste to minimize the length of routes, giving also the routes each truck has to cover. 

The data of the city infrastructures are taken as input (from GIS databases) to represent the city as a graph *G* = (*N*, *E*):

- *E* is the set of roads in the city (or area under study). Each road in the street is denoted as a one-way link *e* ∈ *E*. The length (in km) of each link is denoted as $l_{e},\;\forall e\in E$.
- *N* is the set of intersections among roads. Each intersection is denoted as a node *n* ∈ *N*. ∀ *n* ∈ *N*, there is a set of incoming links *δ^−^(n)* and outgoing links *δ^+^(n)*.
- The location (streets, roads) of those containers with a fill level equal or higher than a threshold is managed as a subset of *E,* denoted as *E’*. Without loss of generality, each link is assumed to have at most one container. Modeling multiple containers is possible by transforming the link into a concatenation of as many links as containers.

Other data are taken as input parameters:

- *T:* maximum number of trucks to use, *t* ∈ {1, 2, …, T}.
- *B*: maximum length in km that a truck is allowed to traverse.
- *S*: cost of using a truck in the route understood as the cost of truck maintenance and driver salary.
- *M*: cost per km travelled by a truck.
- *Z*: collection threshold.
- $p_{e},\;\forall e\in E’$: percentage of waste per container (filling percentage).
- $c_{e},\;\forall e\in E’:\;$container capacity.
- $a_{e},\;\forall e\in E$: maximum number of truck passes allowed in road *e*.
- $C_{t},\;\forall t\in T$: truck capacity.
- *n_s_*∈*N*: origin node of trucks’ routes.
- *n_f_*∈*N*: destination node of trucks’ routes.

The ILP formulation is given as follows:

Decision variables

(1a) $$y_{t}:\;\{\begin{array}{l} 1\;if\;truck\;t\;is\;used\;in\;the\;waste\;collection\\ 0\;otherwise \end{array}\;\forall t\in T$$

(1b) $$w_{te}:\;\{\begin{array}{l} 1\;if\;truck\;t\;collects\;waste\;of\;container\;in\;e\\ 0\;otherwise\; \end{array}\;\forall e\in E’,\forall t\in T$$

(1c) $$x_{te}:\;\{Number\;of\;times\;truck\;t\;traverses\;link\;e\;in\;its\;path\;\forall e\in E,\forall t\in T$$

(1d) $$x_{te'e}:\{\begin{array}{l} 1\;if\;truck\;t\;is\;traversing\;link\;e\;in\;its\;path\\ \;from\;origin\;node\;to\;bin\;in\;e^{\prime },\\ 0\;otherwise \end{array}\;\forall t\in T,\;\forall e\in E,\;\forall e\in E'$$

Objective function

(1e) $$\min\left(M\cdot \underset{te}{\sum }x_{te}\cdot l_{e}+\;S\cdot \underset{t}{\sum }y_{t}\;\right)$$

Constraints

(1f) $$\sum_{e}l_{e}x_{te}\leq \;B\cdot y_{t}\;\forall t\in T,\;\forall e\in E$$

(1g) $$w_{te}\leq x_{te}\;\forall e\in E^{\prime },\forall t\in T$$

(1h) $$\sum_{t}w_{te}=1\;\forall e\in E^{\prime }$$

(1i) $$x_{te’e}\leq x_{te}\;\forall t\in T,\forall e^{\prime }\in E,\forall e\in E$$

(1j) $$\sum_{e\text{Є}\delta^{+}\left(n\right)}x_{te}-\sum_{e\text{Є}\delta^{-}\left(n\right)}x_{te=\;}\{\begin{array}{l} y_{t}\;if\;n=n_{s}\;\\ -y_{t}\;if\;n=n_{f\;}\\ 0\;otherwise\; \end{array}\;\forall n\in N,\;\forall t\in T.\;$$

(1k) $$\sum_{eЄ\delta^{+}\left(n\right)}x_{te'e}-\sum_{eЄ\delta^{-}\left(n\right)}x_{te'e=\;}\{\begin{array}{c} w_{te'}\;if\;n=n_{s}\;\\ -w_{te'}\;if\;n\;=\;b\left(e^{\prime }\right)\\ 0\;otherwise\;\;\;\; \end{array}\;\forall t\in T,\forall e^{\prime }\in E^{\prime },\;\forall n\in N$$

(1l) $$\sum_{e}w_{te}\cdot c_{e}\leq C_{t}\cdot y_{t}\;\forall t\in T$$

(1m) $$\sum_{t}x_{te}\leq a_{e}\;\forall e\in E$$

The ILP is shown in the set of Equation (1). The decision variables *y_t_* (1a) are set to 1 if a truck *t* ∈ *T* is used to collect waste, or 0 otherwise. The decision variables *w_te_* (1b) are set to 1 if a truck *t* collects the waste of container placed in *e* ∈ *E’,* or 0 otherwise. The decision variables *x_te_* represent the number of times a truck *t* goes through a link *e*. 

The ILP is based on the flow-link formulation [29] for modeling the inherent routing problem. A variation of it is presented to avoid solutions with isolated cycles, a possible outcome in regular flow-link formulations consisting of a cyclic path not connected to truck origin or destination node. To forbid such solutions, that are not realizable by a truck in reality, we use two flow-link formulations. The first is represented by *x_te_* variables (1c), which will produce the path followed by each truck *t*. In its turn, *x_te’e_* variables (1d) represent the sub-path of each truck from the origin node, to the end node where each bin is, if the truck is passing through it. The sub-path will have to traverse only streets in the path. That is, a link belongs to a sub-path of the truck, only if the same link is part of the path. 

The objective function (1e) seeks to minimize the cost, given by a term proportional to the distances of the routes of the trucks, plus a fixed cost of each truck just for using it to collect waste. Constraints in (1f) ensure that if a truck *t* is not used to collect the waste, it cannot pass through any link in the network and also permits limiting the total length one single truck can follow (*B*). Constraints in (1g) ensure that if a truck does not pass through a link, it cannot collect the waste of the container placed in that link. Constraints in (1h) ensure that each container with a fill level equal or higher than a threshold will be collected by one truck. Constraints in (1i) mean that if a truck does not pass through a link, no sub-path of the same truck passes through that link. Constraints in (1j) and (1k) are the two sets of flow conservation constraints for the path and sub-path of the trucks. (1j) are the regular flow conservation versions, that define the path of the truck over the streets. Constraints (1k) define the sub-paths. If a truck *t* passes through a link *e*’ with a bin, then there should be a path (described by *x_te’e_* variables) from the truck origin node, to end node of *e*’, using only the streets actually traversed by the truck. Constraints in (1l) limit the maximum amount of waste that each truck can collect. Finally, constraint (1m) permit addressing the acoustic impact in each street: allows to limit the maximum number of times that a truck (summing all the trucks) pass through each street.

Note that the algorithm in Equation (1) takes into account street cuts and rearrangements of street directions since Net2Plan manages the sets *E* and *E’* from the data processed by Net2Plan-GIS plugin, importing updated data from the GIS database of the city. 

## 4. Case Study: Optimal Waste Collection in Cartagena City

This section explains the case study carried out in Cartagena city (Spain), in four subsections. The first one introduces the planning tools used, the second one explains the features of the area under study and how it is managed in the planning tool and the rest presents the features assumed about trucks and waste containers. 

### 4.1. Net2Plan Tool and Net2Plan-Geographical Information Systems (GIS) Extension

**Figure 1:**
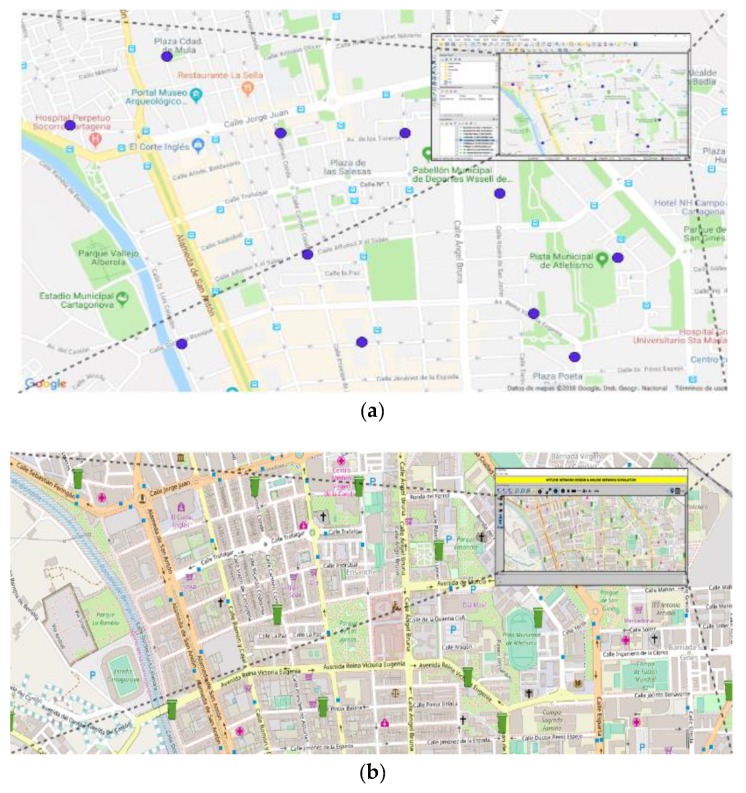
(**a**) Snapshot of QGIS with containers of plastic waste detailed; (**b**) Snapshot of Net2Plan-Geographical Information Systems (GIS) with containers of plastic waste plotted with green icons.

Net2Plan [12] is an open-source and free to use Java-based software designed to overcome the existing barriers imposed by the industry and academia network planning tools for communication networks [11]. After its creation in 2011 and continuous upgrades, Net2Plan has become a powerful network planning software, intended for a broad spectrum of users: industry, research and academia. Its open-source nature makes Net2Plan an ideal tool to develop extensions and plugins when the scenario requires it. Examples of this in urban scenarios are found in [14,30]. Net2Plan-GIS is one of the open-source extensions of the Net2Plan that permits importing data of different layers from GIS databases, making them available for network optimization. In this work Net2Plan-GIS [14] has been used to import infrastructures data (city layouts of streets, intersections and waste containers) from Cartagena city in Spain (see Figure 1), which were stored in a QGIS database [31].

The ILP algorithm has been implemented in Net2Plan [12], making use of the open-source JOM [13] library for interacting with the ILP solver. 

### 4.2. Area under Study: Downtown Cartagena 

**Figure 2:**
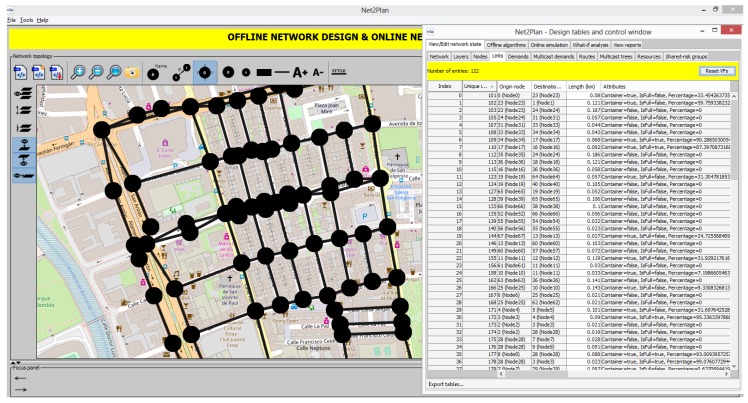
Snapshot of a delimited downtown area of Cartagena in Net2Plan, plotted with nodes and links in intersections and streets respectively. On the right side, the list of links is plotted, with length in km and attributes.

Cartagena is a medium size Spanish city with 220,000 inhabitants. The area under study is a delimited downtown area of 1 km^2^ and around 15,000 inhabitants (see Figure 2). This area is represented as a topology with set of nodes and links *G = (N, E)*. Each link represents a one-way road in the streets. In two-way streets two unidirectional links are set. Each node represents the intersection of two or more streets. A node can also divide those streets with more than one waste container in the way of the street. 

The area under study is a topology composed of 78 nodes (street intersections) joined by 121 unidirectional links which represent the way of streets and the real length of them. In the area under study there are 53 waste containers for plastic. The locations of plastic waste containers are managed by Net2Plan as a Boolean attribute of links, with name Container (see Figure 2). It is set to true if a plastic waste container is located in that street and false otherwise. 

### 4.3. Truck Model for Waste Collection

The truck model Iveco AD260S31 Y/PS [32] has been assumed for the case study. The fuel consumption of this truck is 0.35 L/km not loaded and 0.50 L/km fully loaded. Its maximum payload is 10000 kg and it complies with the Euro 6 regulations for CO emissions: 1.5 g/kWh (2.68 kg/L). See Table 2.

### 4.4. Smart Waste Container 

The waste containers considered in this work are plastic waste containers with 2600 L of capacity, that is, around 100 kg of crushed plastic waste [33]. We assume containers are smart like those in [9,10], with a sensor attached to them (inside them) to measure the filling percentage and a Radio Frequency module with LoRa or GPRS technology to send the data to a server or centralized system. An example of smart device for collecting waste data is 32U4 with MODULE7 GPRS/GSM/GPS, a cheap solution (around €20 per unit) [34] with rechargeable batteries. If a single sending data per day is planned, the batteries life can reach almost one year. The centralized system can be a server connected to Internet, receiving data from smart containers and processing them to update the GIS database of the city. Then, the Net2Plan-GIS and Net2Plan can be used for generating the optimal routes using real and updated data. 

## 5. Formulation Parameters

The ILP searches for the optimal routes in terms of a measure cost combining the variable cost dependent on the route distance and a fixed cost for each truck actually used, whatever distance is traversing. Route distances are directly related to fuel consumption and CO_2_ emissions, two key parameters that must be analyzed. Fuel consumption ($f_{c}$) per truck and time unit (day, week, month) and kg of waste collected, is formulated following equations proposed in [26]. It is calculated as:

(2) $$f_{c}\;=\frac{f_{d}-\left(f_{t,empty}+f_{t,full}\right)}{W}$$

$f_{d}$ is the total fuel consumption. $f_{t,empty}$ is the fuel consumption given by the distance from the garage where the empty truck is parked to the place where the waste collection route starts plus the distance from the landfill, where the truck has dumped the waste collected—and it is empty again—to the garage or to the place where a new route starts, if the truck has to carry out a set of routes the same time unit considered (e.g., three routes in the same day). $f_{t,full}$ is the fuel consumption given by the distance from the last collection in a route, where the truck is full, to the landfill. $f_{d}$, $f_{t,empty}$ and $f_{t,full}$ are measured in litres. Finally, *W* is the amount of waste collected, measured in kg. The fuel consumption per track ($f_{c}$) is measured in litre/kg. 

Fuel consumption per truck and time unit can be also used to get the cost-in any currency-per each kg of waste collected by a truck in the time unit considered. It is calculated as follows:

(3) $$C\;=f_{c}*P_{f}$$

*C* is the cost, measured, e.g., in €/kg and $P_{f}$ is the fuel cost per litre (in this work is set to 0.19 €/L).

CO_2_ emissions ($Eco_{2}$) is calculated using a simplified version of the equation formulated in [27,35]:

(4) $$Eco_{2}=\frac{\sum^{\text{​}}L*EF_{fuel}}{F}$$

*L* is the distance covered by a truck, measured in km. The sum of all distances covered by all trucks is multiplied by $EF_{fuel}$, the CO_2_ emissions factor (kg CO_2_/L) and divided by the fuel consumption index *F* (km/L), given by:

(5) $$F=\frac{\sum^{\text{​}}L}{\sum^{\text{​}}f_{c}*\sum^{\text{​}}W}$$

For sake of simplicity we set $\frac{1}{F}=0.425$, that means that fuel consumption per km is 0.425 L.

$W_{a}$ is the average of total amount of waste collected by those trucks involved in the collection (*R*), measured in kg:

(6) $$W_{a}=\frac{\sum^{\text{​}}W}{R}$$

Finally, the efficiency of a route (*Ef*) is computed as the relationship between the total amount of waste collected in a route and the route length. It is measured in kg/km and it is computed as follows:

(7) $$Ef=\frac{W}{L}$$

## 6. Results

The scenario described in Section 4 is used as a use case example to apply the algorithm and analyze the results. The infrastructure data of a selected area in Cartagena downtown is managed by Net2Plan-GIS. Fifty-three (53) waste containers are candidates to be collected. Each of them can collect up to 100 kg of plastic waste. Since smart containers are not installed in Cartagena city yet, the fill level of garbage in containers has been set randomly, from 1% to 100% of their capacity. Trucks are like those indicated in Section 4.3. Three values of capacity in trucks (amount of waste collected) are considered in the study: 1500 kg, 2600 kg and 6700 kg. 

The algorithm seeks to minimize the cost and shows as output, not only the optimal truck routes (path of streets that trucks have to traverse) but also economic and environmental information (Section 5): amount of CO_2_ emissions, fuel consumption, cost per kg of waste collected, efficiency of the optimal route and noise impact per street (measured as number of times any truck traverses each street). The ILP uses as an input a threshold value which fixes the filling percentage of garbage in the containers, so when the waste in them exceeds it, are selected to be collected and thus included in the algorithm for being visited. Input parameters S (cost of using a truck in the route, understood as the cost of truck maintenance and driver salary) and M (cost per km travelled by a truck) have been set to 100 and 0.19, respectively.

**Figure 3:**
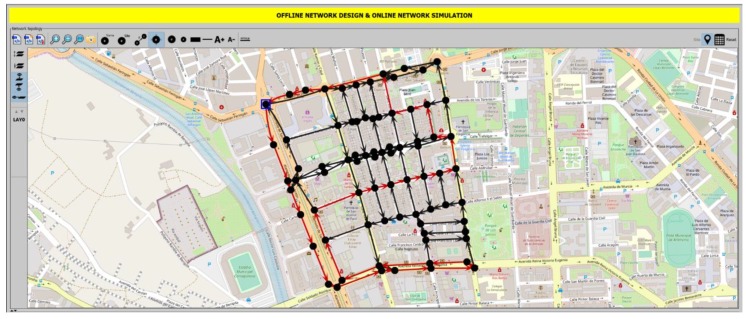
Snapshot of Net2Plan-GIS after integer linear programming (ILP) execution in the scenario under study in Cartagena city, setting threshold of waste level in containers to 85%. Optimal path route in red color.

The scenario has been computed setting as origin and destination nodes in the route nodes 23 and 1 respectively (blue square in Figure 3). The ILP has been ran for different threshold values, from 5% to 95% in steps of 5%. Low threshold values mean that almost all the containers must be collected. High threshold values result in collecting only a few. The results obtained by the ILP are compared with a reference value, given by the real behaviour of truck routes in Cartagena: two trucks with 6700 kg of capacity with fixed routes, visiting between both all containers, independently of their fill level. Each point plotted has been computed one hundred times with different random amounts of waste in the collectors and the average has been extracted. 

As example, Figure 3 shows a snapshot of an output provided by the optimal path problem algorithm executed in Net2plan-GIS in the scenario under study and with a threshold set to 85%. The output (optimal solution) of the ILP is the use of a single truck and the shortest path for that truck, represented in the map as the sequence of red links (streets) that the truck traverses to collect the garbage of all containers with a fill level equal or higher than 85%. Node marked with a blue square is node 23, the origin of the truck routes, while destination node (node 1), is next to it.

**Figure 4:**
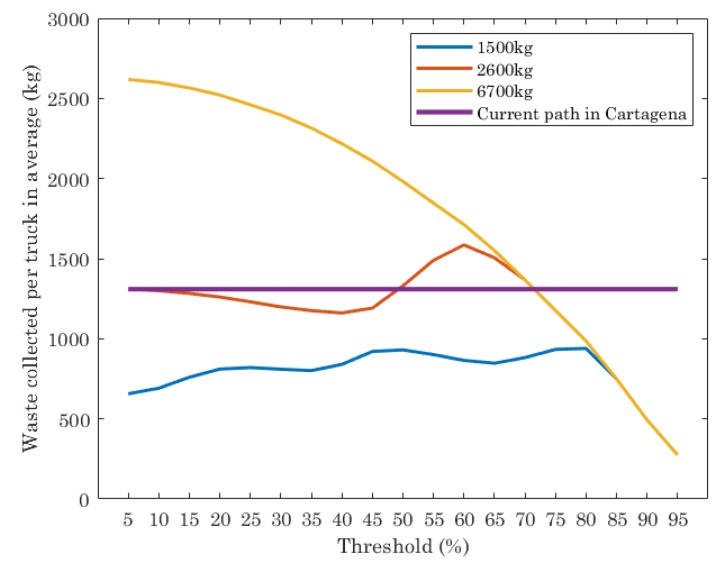
Average waste collected per truck (different truck capacity) for the optimal path given by ILP and the real behavior of truck routes in Cartagena.

Figure 4 shows the results of waste collected (kg) per truck in average, with the fixed route used by the company in charge of collecting waste in Cartagena and the optimal path given by the ILP execution, for different truck capacities and threshold values. Note that the current path in Cartagena is planned with two trucks with maximum capacity of 6700 kg each one and it does not depend on the threshold. Their routes are scheduled with the aim at visiting all containers, independently of the amount of waste in them. On the contrary, the ILP gives as output the number of trucks to use and the optimal path of each one, that depends on the threshold set.

The results of the current path in Cartagena shows that the average of waste collected per truck is almost 1400 kg. The result of the optimal path for trucks with different capacities depends on the threshold set in the ILP execution and truck capacities. For trucks with low capacity (1500 kg and 2600 kg), the waste collected per truck is lower than the current path for almost all thresholds evaluated, while for trucks with 6700 kg of capacity, the waste collected strongly depends on the threshold. For threshold values from 5% to 70%, the average of waste collected per truck is higher than the result provided by the current path. When the threshold is higher than 70%, the amount of waste per truck in average is lower than the current path. 

**Figure 5:**
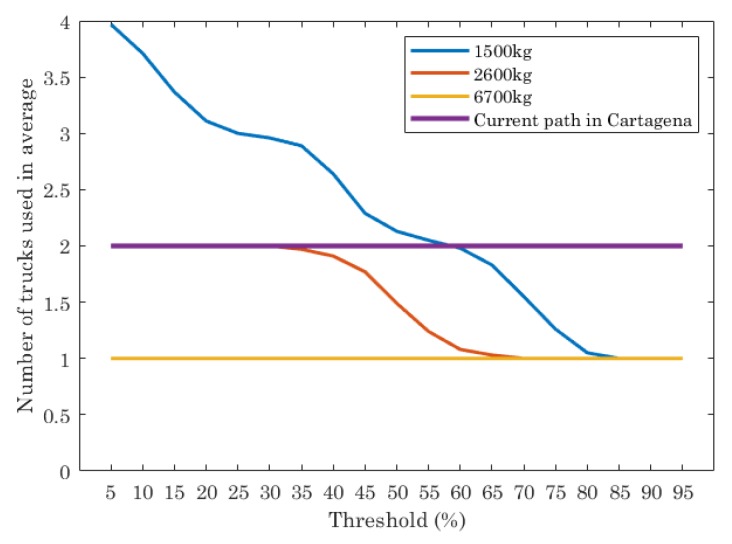
Number of trucks used, in average, for the optimal paths given by ILP and the current path in Cartagena.

In order to shed some light to the previous results, Figure 5 is plotted. It shows the number of trucks used in average for different truck capacities, as well the data for the current path (two trucks). The average is calculated taking into account that each scenario has been executed 100 times. Each point in the graphic is the average of these results. 

As can be seen, the results of the ILP show that the truck capacity and threshold impact on the number of trucks used. If trucks with low capacity (1500 kg) are considered in the ILP and the threshold is set to 5% (almost all containers must be emptied), then the number of trucks to use for traversing the optimal paths is 4. As the threshold increases, the number of trucks needed decreases, being lower than the current path when the thresholds are 65% or higher. If trucks of 2600 kg of capacity are used, the ILP shows that the optimal path needs two trucks when threshold is set between 5% and 35%. When the threshold is [35%, 65%], the number of trucks needed is between 2 and 1. Finally, when the threshold value is higher than 65%, the number of trucks needed is always 1. When trucks are 6700 kg of capacity the ILP execution gives as a result that only one truck is the optimal choice, independently of the threshold. 

**Figure 6:**
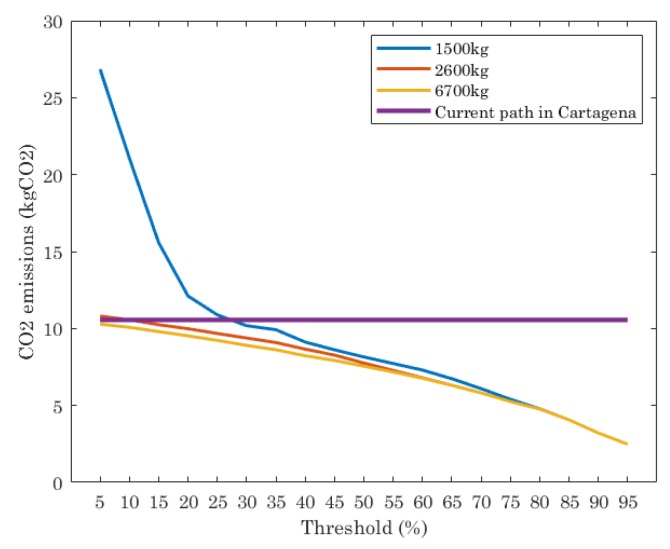
Amount of CO_2_ emissions: for the optimal paths given by ILP and the current path in Cartagena.

The number of trucks, the amount of waste they collect and the length of the routes are directly related to the CO_2_ emissions. This parameter was formulated in Section 5. Figure 6 shows the results of this in the scenario under study for the current path in Cartagena and the ILP executed for different truck capacities and thresholds. From Figure 6 we see that the current path has a constant value of CO_2_ emissions, since it is a fixed route with a fixed number of trucks, independently of the threshold value. The ILP shows that, depending on the threshold and truck capacity, the CO_2_ emissions varies. When trucks with 1500 kg of capacity are considered, the CO_2_ emissions are strongly dependent on the threshold. Threshold values in range [5%, 25%] means CO_2_ emissions higher than the current path. When threshold is set to 25% or lower the ILP reports lower CO_2_ emissions than the current path.

When trucks have a capacity of 2600 kg and 6700 kg, the CO_2_ emissions given by the optimal path are similar in all of thresholds evaluated and lower than the current path. Only when the threshold is set to [5%, 15%] and trucks are of 2600 kg of capacity, the ILP reports similar values of CO_2_ emissions than the current path. 

**Figure 7:**
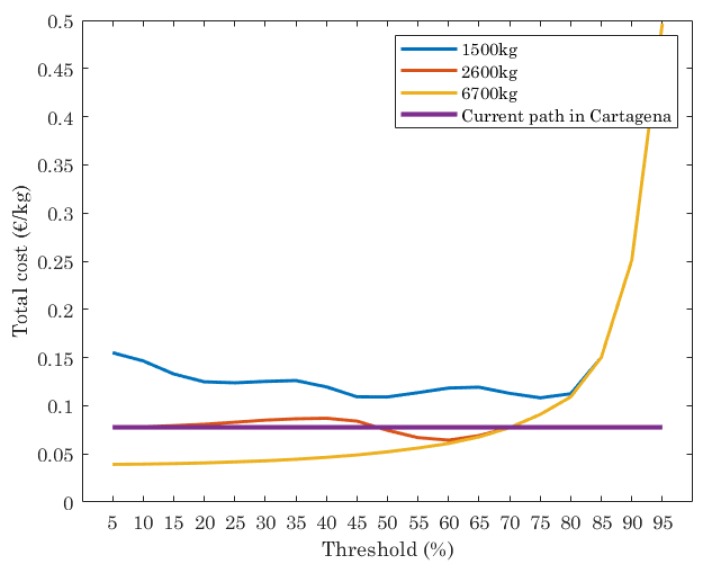
Cost (€/kg), for the optimal paths given by ILP and the current path in Cartagena.

Finally, the total cost of waste collected (€/kg) is shown in Figure 7. This depends on the length of the paths computed (that impacts also CO_2_ emissions) and by the number of trucks used, with a fixed cost per truck (€100), which includes cost of truck maintenance and driver salary per day. First thing to note is that total cost is, in our case study, dominated by the fixed cost of the trucks, that have a much higher impact in the final cost than the route lengths. Results plotted in Figure 7 show that the current path has a constant cost, while the ILP reports higher cost when trucks with 1500 kg of capacity are assumed, since more trucks are needed. When trucks with 2500 kg of capacity are used, the cost is almost equal than the current path, independently of the threshold set. However, if the trucks have 6700 kg of capacity, the cost is lower than the current path when the threshold is set in the range [5%, 70%].

From these results, we can conclude that the optimal path for waste collection strongly depends on the input parameters. For this study, we evaluate the ILP for different values of capacity of trucks and threshold. These have a direct effect in the amount of waste collected and number of trucks used, un thus, CO_2_ emissions and cost of waste collection. For the scenario under study, the best performance in terms of waste collected, cost of waste collection and CO_2_ emissions, is achieved when trucks have 6700 kg of capacity and the threshold is set in the range [5%, 60%]. When the threshold is in the range [60%, 95%] it is better to use trucks with 2600 kg. 

## 7. Conclusions and Future Work

In this work, an optimal path planning algorithm based on an ILP (integer linear program) has been presented, with the capability of jointly deciding the number of trucks to use and their optimal routes and with the possibility of considering constraints like acoustic impact in the streets, or maximum route lengths for each truck. The algorithm is integrated into the open-source Net2Plan-GIS planning tool, which facilitates the application of this algorithm in practical use cases, in particular, allowing the input of data from GIS databases. In addition, the presented framework provides performance merits like fuel consumption, CO_2_ emissions and other economic and environmental figures. As an added value, our framework can export the path data into Google Maps, making it a powerful tool for smart cities.

To showcase our proposal, we present a realistic use case assessing the waste collection process in Cartagena city (Spain). The algorithm uses as input data from the GIS databases of the city and data received from smart containers (e.g., filling percentage). In the case study, our proposed algorithm assists the process of evaluating the benefits of using smart bins and optimizing the fill level threshold in the smart bin that should drive the decision on when to collect a bin.

## Figures and Tables

**Table 1:** Comparison of the main features of algorithms reviewed in state of art.

Ref.	Algorithm	Goal	Smart Bins	GIS	Street (≥1)	#Trucks as Output
**[9] **	Different heuristics and local search method	Minimize Distance	√	√	x	X
**[20] **	MILP	Minimize Distance	x	x	x	X
**[21] **	Heuristic: Nearest Neighborhood	Minimize Distance	√	√	x	√
**[22] **	Meta-heuristic: AnColony Optimization	Minimize Distance	x	x	x	√
**[23] **	Meta-heurtic: Genetic algorithm	Minimize Distance	x	√	x	X
**[24] **	Meta-heuristic: Chaotic Particle Swarm Optimization	Maximize total waste collected	x	√	x	√
**[25] **	Meta-heuristic: Tabu search	Minimize transport cost + maximize service quality	x	x	x	X
**[27] **	Meta-heuristic: Backtracking Search Algorithm	Minimize Distance	√	x	x	√
**[28] **	Meta-heuristic: Particle Swarm Optimization	Minimize Distanc	√	x	x	√
***	ILP	Minimize Distance + Truck/s cost	√	√	√	√

**Table 2:** Euro emissions (g/kWh).

Type	CO	HC	NOx	PM
Euro VI	1.5	0.13	0.4	0.01
